# Supplementary material for: Reduced-Beclin1-Expressing Mice Infected with Zika-R103451 and Viral-Associated Pathology during Pregnancy
Source: Viruses. 2020 Jun 2;12(6):608. doi: 10.3390/v12060608 (PMC7354588; doi:10.3390/v12060608)
Supplement: Supplementary file 1 [file viruses-12-00608-s001.pdf]

Reduced-Beclin1-Expressing Mice Infected with Zika-R103451 and Viral-Associated Pathology during Pregnancy

Mohan Kumar Muthu Karuppan[1], Chet Raj Ojha[1], Myosotys Rodriguez[1], Jessica Lapierre[1], M. Javad Aman[3], Fatah Kashanchi[2], Michal Toborek[4], Madhavan Nair[1] and Nazira El-Hage[1*].

1 Department of Immunology and Nanomedicine, Herbert Wertheim College of Medicine, Florida International University, Miami, FL 33199, USA
2 National Center for Biodefense and Infectious Diseases, George Mason University, Manassas, VA, 20110, USA
3 Integrated biotherapeutics, Rockville, MD, 20850, USA
4 Department of Biochemistry and Molecular Biology, University of Miami Miller School of Medicine, Miami, FL 33136, USA.
* Correspondence: Dr. Nazira El-Hage, Department of Immunology and Nanomedicine, Florida International University, Herbert Wertheim College of Medicine, Miami, FL 33199, USA; E-mail address: nelhage@fiu.edu; Phone: (305)-348-4346; FAX: (305)-348-1109.

SUPPLEMENTAL MATERIAL

MATERIALS AND METHODS

Immunohistochemistry

ZIKV infectivity was measured by fluorescent immuno-labeling. Briefly, brain tissue sections were fixed in 4% paraformaldehyde, permeabilized with 0.1% Triton X-100, and blocked in 10% milk/0.1% goat serum. The sections were then immunolabeled with the neuronal marker, mouse MAP2 (microtubules associated protein-2) antibody (Catalog# MAB378; Millipore, Boston, MA, USA), ZIKV-E antibody (Catalog# GTX133314) and ZIKV-NS1 antibody (Catalog# GTX133307) purchased from Genetex, CA, USA. Immunoreactivity was visualized with secondary antibodies from Molecular Probes (Carlsbad, CA, USA). Cell nuclei was labeled with 4′,6-diamidino-2-phenylindole (DAPI). The images were analyzed using an inverted fluorescence microscope with a 560 Axiovision camera (Zeiss, Germany).

MTT assay

Glia were grown for 24 h in their respective culture media with increased concentrations of viral proteins. Media was exchanged with protein-free medium containing 0.5 mg/mL MTT, and cells were further

incubated for 3 h. The MTT-containing medium was removed, and 100 μL of DMSO was added to solubilize the formazan. The absorbance at 570 nm was measured to determine viability using a Synergy™ H4 Hybrid Microplate Reader (BioTek Instruments, Inc. Winooski, VT).

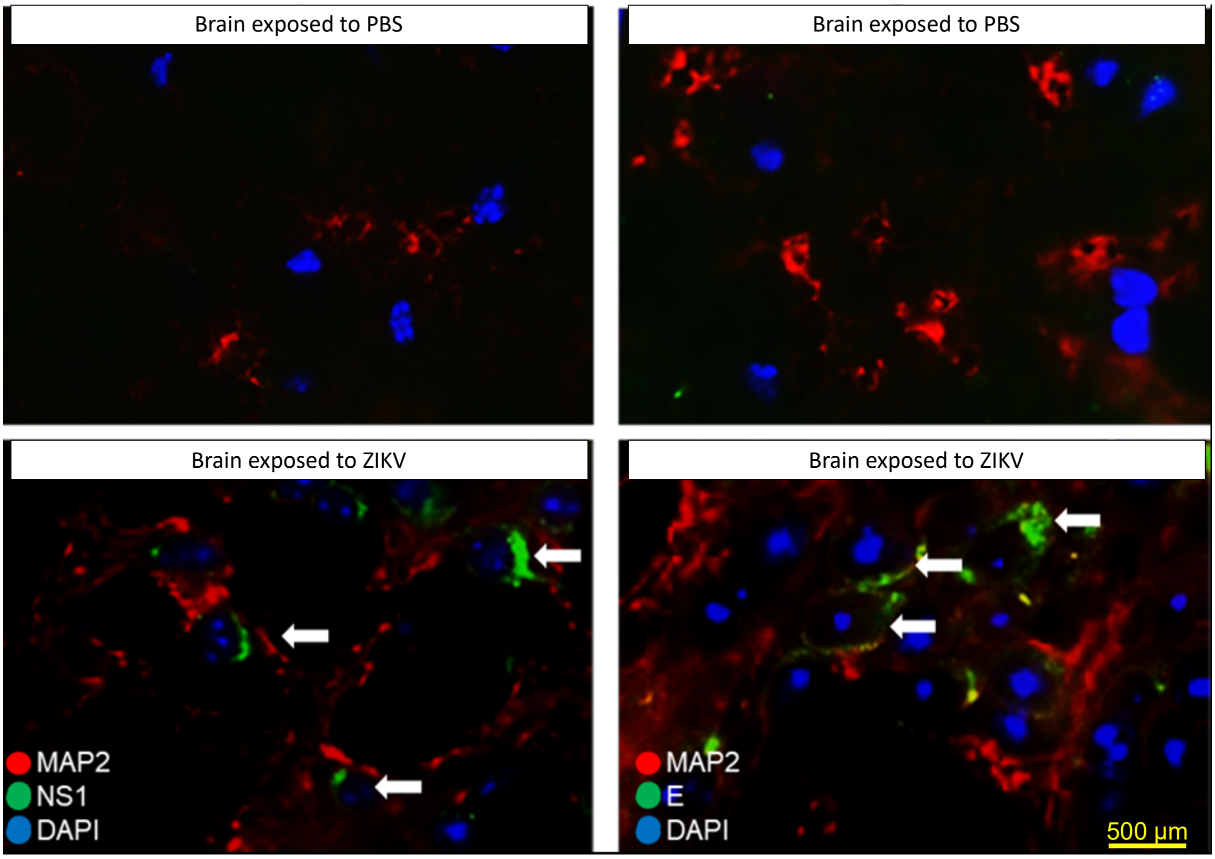

Figure S1: Detection of viral proteins in post-mortem brains. Immunofluorescent double labeling with antibodies against the neuronal marker, MAP2 (shown in red) and ZIKV proteins (shown in green). Expression levels of NS1 (left bottom panel) and the structural E protein (right bottom panel) are shown in brain tissues recovered from *Becn1*$^{+/-}$ pups born to ZIKV-infected dams. Brain tissues recovered from *Becn1*$^{+/-}$ pups born to mock-exposed dams showed no fluorescent labeling with NS1 or E antibodies (top panels).

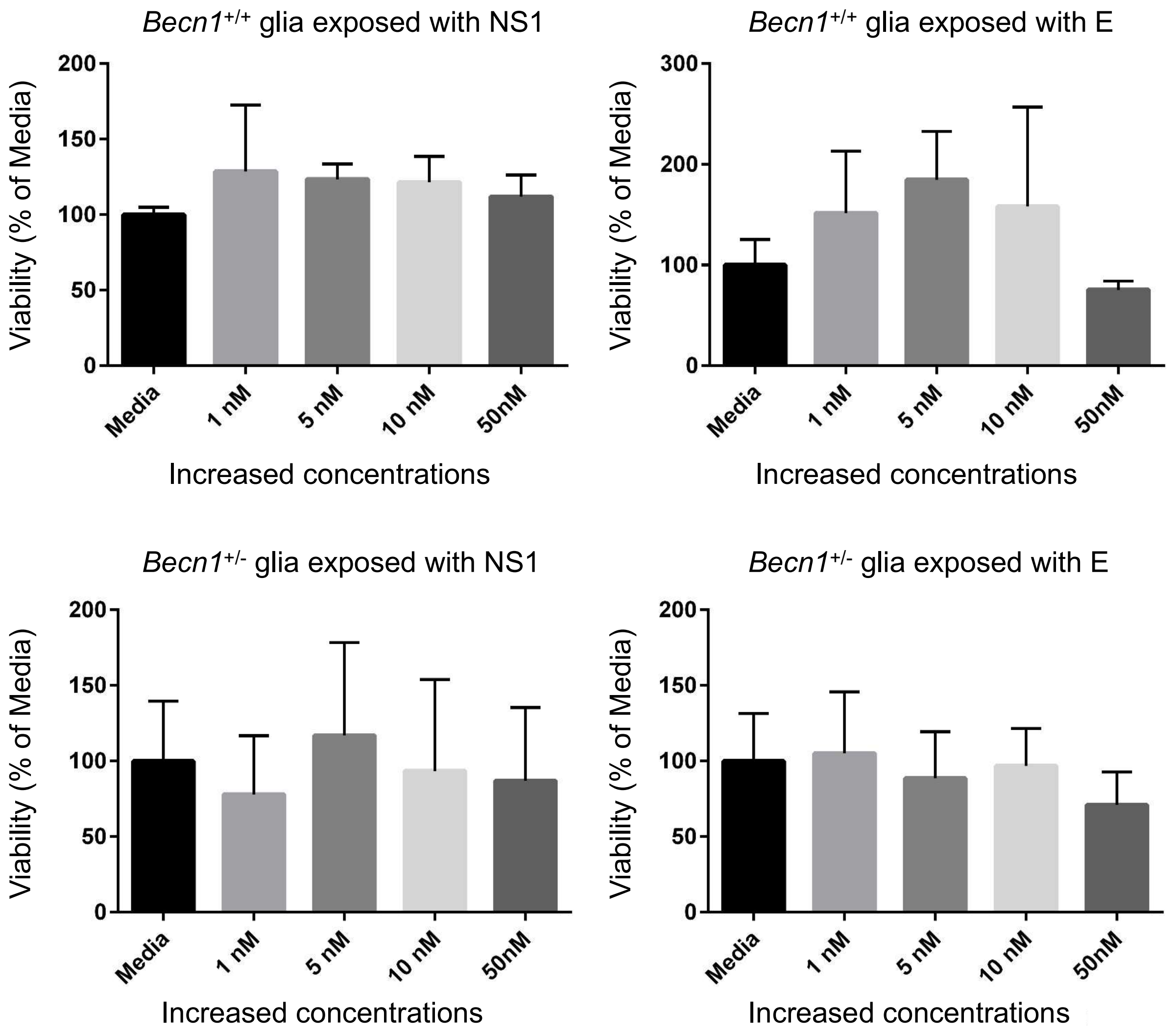

Figure S2: **Dose-response curve of viral proteins**. Cell viability was assayed after 24-hours post-treatment by the ability of live cells to reduce the tetrazolium dye 3-(4,5-dimethylthiazol-2-yl)-2,5-diphenyltetrazolium bromide (MTT) to purple formazan. NS1 (left panels) and E (right panels) proteins showed minimal toxicity to glia recovered from *Becn1*$^{+/+}$ (top) and *Becn1*$^{+/-}$ (bottom) pups. Proteins at 50nM concentrations were subsequently used to treat murine glia cultures.
